# CN metabolism and nitrogen use efficiency of rice with different nitrogen form and rate

**DOI:** 10.1371/journal.pone.0318522

**Published:** 2025-03-25

**Authors:** Minji Kim, Boyun Lee, Jwakyung Sung

**Affiliations:** Department of Crop Science, Chungbuk National University, Cheongju, South Korea; Osmania University, INDIA

## Abstract

Nitrogen (N) is one of crucial mineral nutrients for rice cultivation, however excessive N application has resulted in lower utilization and thus occasionally attributes to environmental impacts. Simultaneously, rice production requires greater watering, exacerbating water scarcity concerns. This study explores strategies to enhance nitrogen use efficiency (NUE) in rice, focusing on the carbon-nitrogen (CN) metabolism under different nitrogen conditions. Two rice cultivars (*Oryza sativa* L. cv. Samgwang-SG, and NIL Milyang#360-ML) were subjected to different nitrogen forms (ammonium sulfate-AS, ammonium nitrate-AN) and application rates (45 kg ha⁻¹ and 90 kg ha⁻¹). The results demonstrated that SG exhibited increased N assimilation in both leaves and roots under lower N input, while ML primarily superior on grain development. ML showed higher carbohydrate accumulation in leaves, potentially contributing to enhanced grain yield under low N conditions. Moreover, ammonium-sulfate (AS) proved more effective in promoting NUE than ammonium-nitrate (AN), particularly at lower N input (45N). Principal component analysis confirmed that 45N treatments positively correlated with improved nitrogen uptake and utilization efficiency, with no significant yield reduction. These findings highlight the importance of optimizing nitrogen management to improve NUE while reducing environmental impacts in rice production. A further study is required to evaluate and validate the nitrogen use efficiency under different N form and dose with a field scale.

## Introduction

Nitrogen (N) is a crucial component of amino acids, nucleic acids, chlorophyll, and phytohormones, making it a key factor in the growth and development of crop plants. Typically, only about 30-40% of applied nitrogen is utilized by plants, which drives the effort to enhance nitrogen use efficiency (NUE) through better crop breeding strategies and agricultural practices [1,2]. Despite this reason, farmers often increase nitrogen input to ensure high rice production [3,4], though excessive and inappropriate N managements can lead to environmental concerns, such as greenhouse gas emissions. Although ammonium sulfate (AS) contains a lower N content (21% N), it offers agronomic and ecological benefits, including reduced NO3-N leaching, enhanced N efficiency, and lower greenhouse gases (NO and N2O) [5]. The NUE is determined by N uptake, transport, assimilation and remobilization, which involve key enzymes like nitrate/ammonium transporters (NRTs/AMTs), nitrate/nitrite reductase (NR, NiR), glutamine synthetase (GS) - glutamine-2-oxoglutarate aminotransferase (GOGAT), asparagine synthetase (ASN) and glutamate dehydrogenase (GDH) [6]. Rice root morphology and physiology are closely associated with N uptake and utilization, as roots regulate metabolic processes, with amino acids being key metabolites in these pathways [7–11]. Nitrogen assimilation primarily occurs in leaves for photosynthesis and tillering during the vegetative stage, later contributing to amino acid synthesis for grain development [12,13]. Roots play a dual role in nutrient acquisition and amino acid metabolism [14,15], with low N conditions promoting root development and lateral root differentiation to improve N availability, while excessive N supply inhibits root growth [16–18].

Rice, a staple food crop for over half of the world’s population, faces increasing demand, projected to rise by 44% by 2050 [19]. Recent studies, such as Kwon et al [20], have reported that the *gs3* mutant and low N input in a near-isogenic line (NIL) Milyang#360 (backcross hybrid of Saeilmi and Shindongjin) reduced methane emissions after the heading stage, enlarged grain size and enhanced NUE. Comparative genetic variations of NUE in crops have been explored in greenhouse and field conditions, with a focus on carbon-nitrogen (CN) metabolism in shoots and roots from seedling to reproductive growth stages [21–25].

Given that leaves are a major site that supplies carbon- and nitrogen-containing metabolites, which determine physiological efficiency, we hypothesized that two contrasting rice cultivars (low or high N-efficient) would exhibit different responses in CN metabolism and NUE. To test this hypothesis, we employed different nitrogen forms (ammonium-nitrate, AN; ammonium-sulfate, AS) and rates (45 and 90 kg ha^−1^) to compare CN metabolism at the heading stage and NUE at the harvesting time. Our results provide valuable insights into nitrogen fertilization strategies for N-saving rice production systems.

## Materials and methods

### Plant material and growth condition

The study was carried out in a greenhouse at Chungbuk National University, Cheonju, South Korea (36°37’48.1“N 127°27’05.4” E) from June to September 2022. Two rice cultivars, Samgwang (SG, normal nitrogen-efficient) and Milyang#360 (ML, high nitrogen-efficient), were used for this experiment. The seeds were germinated for two days in an incubator (25 °C, in darkness), and seedlings were grown in a growth chamber (200 μmol m^−2^ s^−1^ photosynthetic photon flux density under 12-hour photoperiod, relative humidity of 60% and day/night temperature of 25/20 °C) until they reached the 3^rd^ to 4^th^-leaf stage. The seedlings were then transplanted into containers (1/20,000 ha) filled with sandy loam soil characterized by a pH of 4.91, an electrical conductivity (EC) of 0.06 dS/m, 0.64% soil organic matter (SOM), 74.5 mg kg^−1^ inorganic N, 389 mg kg^−1^ soluble K, 458 mg kg^−1^ soluble Ca, and 131 mg kg^−1^ soluble Mg. The rice plants were fertilized with nitrogen (N) at two rates: low N (45 kg ha^−1^) and optimal N (90 kg ha^−1^). In addition to nitrogen, standard doses of phosphorus (P_2_O_5_, 45 kg ha^−1^) and potassium (K_2_O, 57 kg ha^−1^) were applied. The nitrogen fertilization was split into three doses: 50% applied at basal (before transplanting), 30% at the tillering stage (30 days after transplanting, DAT), and 20% at the panicle initiation stage (60 DAT). Two nitrogen forms, ammonium-sulfate (AS) and ammonium-nitrate (AN), were applied with nitrogen equivalent to 90 or 45 kg ha^−1^. P_2_O_5_ (fused superphosphate) was fully applied before transplanting, while K₂O (potassium chloride) was split, with 70% applied at basal and 30% at the panicle initiation stage. Water was maintained at 2-3 cm above the soil surface throughout the experiment. Each treatment was arranged in a completely randomized design with three replicates.

### Growth parameter and photosynthetic measurements

Plant height, tillers and dry weight of rice plants were measured at the heading stage. Photosynthetic traits were measured on the uppermost fully expanded leaves at the heading stage using a portable photosynthesis system (LC-PRO+ , ADC BioScientific Ltd, Hertfordshire, UK). Measurements were taken between 10:00 AM and 12:00 PM at ambient CO_2_ concentration (400-410 mmol m⁻^3^) under a light intensity of 1300-1600 μmol m^−2^ s^−1^ photosynthetic photon flux density, and an ambient temperature of 30-32 °C. The photosynthetic rate (P_*n*_), stomatal conductance (g_*s*_), transpiration rate (E), and intercellular CO_2_ concentration (C_*i*_) were measured.

### Soluble sugar and starch determination

Soluble sugar and starch were measured in leaves and roots, which were taken at the heading stage. Soluble sugar and starch were measured in leaf and root samples. Powdered samples (0.2 g, fresh weight) were extracted with 10 mL of 80% ethanol and evaporated. Residues were dissolved in distilled water, mixed with 2 volumes of 0.2% anthrone in a concentrated H2SO4, and carbohydrate content was estimated spectrophotometrically at 630 nm (UV-1900i, SHIMADZU, Japan) [26]. Glucose was used as a standard.

### RNA extraction and quantitative real-time PCR analysis

Total RNA extraction using TRIzol reagent (Invitrogen, Carlsbad, CA) according to the manufacturer’s instructions was extracted from leaf blades and roots of rice treated with different form and rate of N fertilization at the heading time. The purity and concentration of the extracted RNA were estimated using NanoDrop (Thermo Fisher Scientific, Madison, WI, USA), and checked on 1.0% agarose gel. Total RNA (1 μg) and RT PreMix Kit (iNtRON Biotechnology, Inc., Seongnam, Korea) with Oligo (dT) primers were used to synthesize first-strand cDNA using the following PCR conditions; 60 min at 45 °C to cDNA synthesis and 5 min at 95 °C to Rtase inactivation step. Quantitative real-time PCR was performed by using a Real-Time PCR machine (CFX Opus 96, Bio-Rad, Hercules, CA, USA) with technical triplicates with the manufacturer’s instructions. The reaction mixture consisted of 1 μL of cDNA template, 2 μL each of 10 mM forward and reverse primer (Table 1), and 5 μL SYBR Green Q Master mix (Labopass, Cosmo Genetech, Seoul, South Korea). The PCR conditions consisted of pre- denaturation step at 95 °C for 3 min, followed by 40 cycles of denaturation at 95 °C for 15 s, annealing temperature of each primer (Table 1) for 15 s, and elongation (72 °C, 15 s). This step was followed by a melting curve, ranging from 65 to 95 °C at a heating rate of 0.5 °C/s. A quantification method (2^–∆∆Ct^) was used [27] and the variation in expression was estimated using triplicate for each cDNA sample. The rice actin gene was used as a reference in the qRT-PCR. Primer sequences used for qRT-PCR were designed by Primer 3 software [28].

**Table 1:** Primers information used for qRT-PCR analysis of nitrogen metabolism genes in rice.

Function	Gene symbol	Tissue	Description	Locus ID	Sequence (‘5 → ‘3)	Annealing Tm (°C)
Ammonium uptake	*OsAMT1;2*	Root	Ammonium transporter1;2	*Os04g0509600*	FW: ACGTCATCCAGATCCTGGTC RV: AGACTTGTCGTGCTCGTCCT	59.0
	*OsAMT2;1*	Root	Ammonium transporter2;1	*Os05g0468700*	FW: GGTGCTCTTCCAGTTCGAGT RV: GACGGTGTAGGAGAGGAGGA	59.0
Nitrate uptake	*OsNRT2;1*	Root	Nitrate transporter2;1	*Os02g0112100*	FW: TACGCCGTCACCAACTACC RV: GGTCGAAGCGATCGTAGAAG	56.0
	*OsNRT1.1b*	Root	Nitrate transporter1.1b	*Os10t0554200*	FW: GTCACCATCGTCCACAAGG RV: GAAGAGGACGAGGTTGATGG	56.0
Assimilation	*OsNiR*	Root, Leaf blade	Nitrite reductase	*Os01t0357100*	FW: ATCAACGACCTCGCGTACAT RV: GAGAACGGCCTTGCACAC	56.0
	*OsNR2*	Root, Leaf blade	Nitrate reductase2	*Os02t0770800*	FW: AGGGTAGAGGTGACCCTGGA RV: GCTGGGTGTTGAGGGACTC	56.0
	*OsGS1;2*	Root	Glutamine synthetase1;2	*Os03g0223400*	FW: ACGGAGAAGGAGGGCAAG RV: CCACAGCAGCGTGGTCTC	59.0
	*OsNADH-GOGAT1*	Root	NADH-glutamate synthase1	*Os01t0681900*	FW: GGCCTGGTCGATTTTATGTG RV: GTGCCTTCAATGCTTCATCA	53.0
	*OsAS1*	Root	Asparagine synthetase1	*Os03t0291500*	FW: CTGAGCTCCATTCCTTCGTC RV: CGTCTCGTCGTGGTAGATGA	55.3
	*OsAAT2*	Root	Aspartate aminotransferase2	*Os01t0760600*	FW: CCCGTCACGTGTTAAGGAG RV: CCTCCCACCCTTAAAGAACC	56.9
	*OsGS1;1*	Leaf blade	Glutamine synthetase1;1	*Os02t0735200*	FW: CTGTGGTATCGGTGCTGACA RV: ATGACCTCGCCGTTGATTC	56.0
	*OsFd-GOGAT*	Leaf blade	Ferredoxin-glutamate synthase	*Os07t0658400*	FW: ACCCTATCAAGTCCTGTGCTT RV: AGCAGCATCAGCTTCATCAC	56.0
	*OsAS2*	Leaf blade	Asparagine synthetase2	*Os06t0265000*	FW: AGGTGGCCTAAGGAGATGGT RV: TCCAGTCCACCAGACAAGAG	50.3
	*OsAAT1*	Leaf blade	Aspartate aminotransferase1	*Os02t0797500*	FW: GGTGAAGAGCCAGCTGAAAC RV: CACTGTCGTCCTTTGCAGAC	59.0
	*OsACTIN1*			*Os03g0718100*	FW: TGTATGCCAGTGGTCGTACC RV: CCAGCAAGGTCGAGACGAA	

### Assessment of nitrogen use efficiency

Total N content was determined from shoot and grain at the harvesting time using a C/N analyzed (VarioMax CN Analyzer, Elementar, Germany). NUE was evaluated using the N content and dry weight from the shoots and grains at the harvesting time, and followed by the equations [29].

1. Nitrogen use efficiency (NUE) = Total biomass (shoot + grain) (g)/Applied N (g)
2. Nitrogen uptake efficiency (NUpE) = Absorbed N (g)/Applied N (g)
3. Nitrogen utilization efficiency (NUtE) = Total biomass (shoot + grain) (g)/Absorbed N (g)
4. Nitrogen use efficiency of grain (gNUE) = Grain (g)/Applied N (g)
5. Nitrogen harvest index (NHI, %) = Grain N accumulation/Total N accumulation (Shoot + Grain)

### Statistical analysis

Data were analyzed using RStudio (Ver. 4.1.3) with one-way ANOVA followed by Tukey’s HSD test if p < 0.05. T-test was used to compare differences between cultivars. Principal component analysis (PCA) was performed to assess the relationship between NUE variables.

## Results

### Growth parameters under different N forms and rates

**Fig 1:**
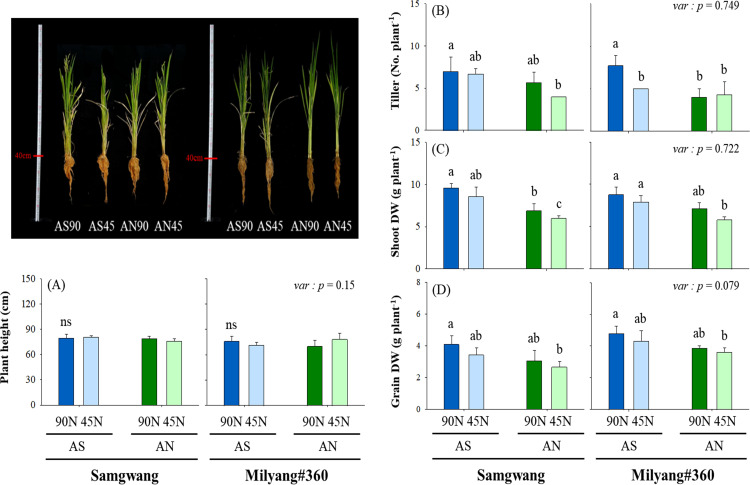
Comparison of plant height, tillering and dry weight (shoot and grain per plant). Nitrogen form is ammonium-sulfate (AS) and ammonium-nitrate (AN), and application rate was 45 (50%) and 90 kg ha^−1^ (100%, standard fertilization rate). Mean difference between treatments was employed with Tukey’s test, if <  0.05, and T-test was used for the difference between cultivars.

In this study, we investigated the effects of different nitrogen forms (ammonium-sulfate, AS, ammonium-nitrate, AN) and rates (45 and 90 kg ha^−1^) on the growth of two contrasting rice cultivars: Samgwang (SG) and Milyang#360 (ML). A difference in plant height and tillering at the heading time point was not observed between N rates, N forms, and cultivars except significantly much more in ML-AS-90N (7.7/plant) compared to ML-AS-45N (5.0/plant) (Fig 1A–B).

The dry weight aboveground at the harvesting time point was not significantly different in all treatment groups except for the SG treated with AN (45N vs. 90N) (Fig 1C). Especially, reduction in grain dry weight was 17% (AS) and 13% (AN) in SG, and 10% (AS) and 7% (AN) in ML (Fig 1D). Accordingly, these results suggest that, ML utilizes N more efficiently than SG, and AS, as an N form, is effective for rice growth compared to AN.

### Photosynthesis and soluble carbohydrates under different N forms and rates

**Fig 2:**
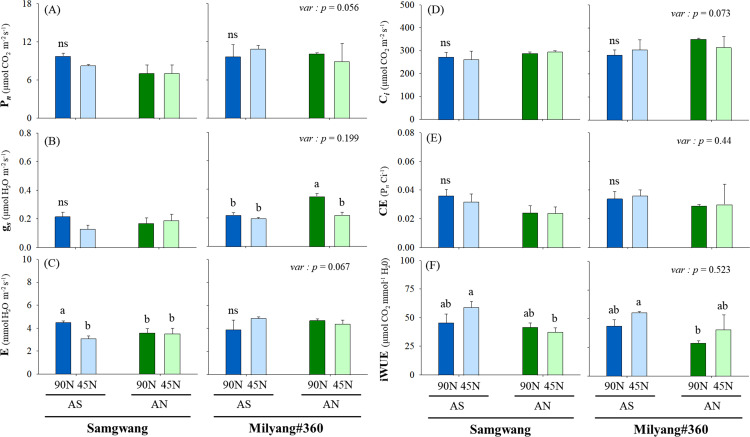
Gas exchange parameters (P_*n*_, *g*_*s*_, E) and water use efficiency. Nitrogen form is ammonium-sulfate (AS) and ammonium-nitrate (AN), and application rate was 45 (50%) and 90 kg ha^−1^ (100%, standard fertilization rate). Mean difference between treatments was employed with Tukey’s test, if <  0.05, and T-test was used for the difference between cultivars.

Gas exchange parameters including photosynthetic rate (P*n*), stomatal conductance (g*s*) and transpiration (E) were measured at the heading time for both cultivars treated with different N forms and rates. Photosynthetic traits were also not different from two cultivars (Fig 2A–F).

**Fig 3:**
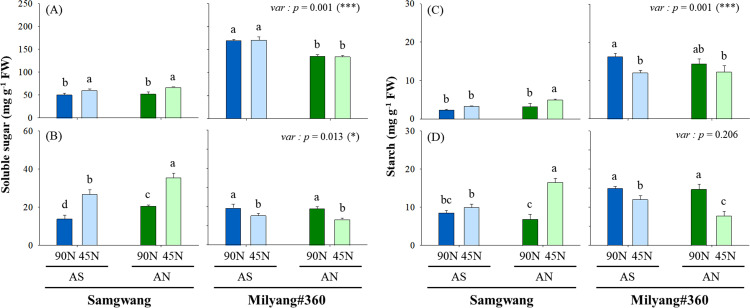
Soluble sugar and starch concentration in leaves and roots. Nitrogen form is ammonium-sulfate (AS) and ammonium-nitrate (AN), and application rate was 45 (50%) and 90 kg ha^−1^ (100%, standard fertilization rate). Mean difference between treatments was employed with Tukey’s test, if <  0.05, and T-test was used for the difference between cultivars.

However, the photosynthetic rate (P*n*) in SG was 28% higher with AS (8.3-8.8 µmol m^−2^ s^−1^) compared to AN (7.0-7.1 µmol m^−2^ s^−1^), while the effect of N form and rate was not displayed in ML. Stomatal conductance (g*s*) was highest in ML-AN-90N (0.35 µmol m^−2^ s^−1^), and reduced N application rates resulted in a decline in g*s* (0.13-0.20 µmol m^−2^ s^−1^). Similarly, transpiration (E) notably increased in SG-AS-90N (4.5 mmol m^−2^ s^−1^) compared to SG-AS-45N (3.1 mmol m^−2^ s^−1^). To evaluate CO2 and water use efficiency, carboxylation efficiency (P*n*/C*i*) and intrinsic water use efficiency (iWUE) was estimated (Fig 2E–F). Carboxylation efficiency showed higher value (0.03-0.04) in AS groups compared to AN in SG, whereas it was not different between treatment groups (0.3-0.4) in ML. The iWUE was also not significant between treatment groups in both cultivars, however AS treatment groups indicated 1.42 times greater (43.5-65.6 µmol CO2 mmol H2O-1) compared to AN (28.9-41.6 µmol CO2 mmol H2O-1). Soluble sugar and starch were measured from leaf blades and roots of SG and ML under different N forms and rates at the heading time (Fig 3A–D).

Soluble sugar was markedly abundant in leaf blades of ML (2.62 times greater, 152.6 mg g^−1^ FW) compared to SG (58.1 mg g^−1^ FW), whereas SG roots (1.44 times, 24.2 mg g^−1^ FW) showed greater than ML (16.8 mg g^−1^ FW) (Fig 3A–B). The AS promoted an accumulation of soluble sugar in the leaf blades of ML, whereas AN made plentiful in the roots of SG. A low N application rate (45N) resulted in the contrasting abundance in roots by cultivars, which indicated greater in SG, meanwhile less in ML, and also stimulated a greater level in leaf blades of SG. Overall, starch concentration revealed a similar pattern with soluble sugar (Fig 3C–D), which indicating that ML showed significantly greater (13.8 mg g^−1^ FW, p<0.0001) in leaf blades compared to SG (3.4 mg g^−1^ FW), while were similar levels in roots (10.5 mg g^−1^ FW for SG; 12.4 mg g^−1^ FW for ML).

### N metabolism under different N forms and rates

**Fig 4:**
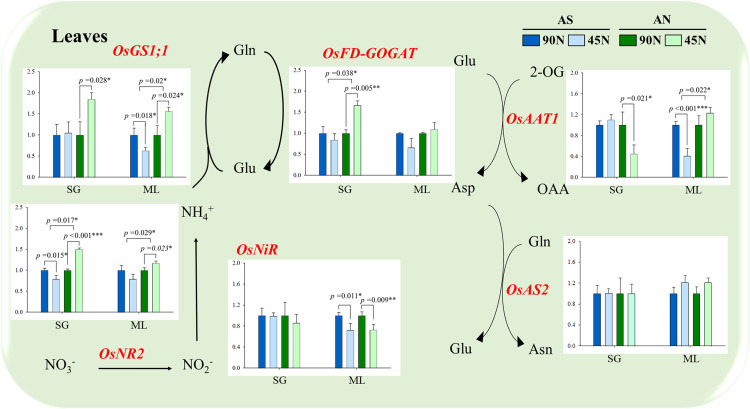
Expression of nitrogen-related genes in leaf blades. Nitrogen form is ammonium-sulfate (AS) and ammonium-nitrate (AN), and application rate was 45 (50%) and 90 kg ha^−1^ (100%, standard fertilization rate). Mean difference between treatments was employed with Tukey’s test, if <  0.05, and T-test was used for the difference between cultivars.

To assess nitrogen metabolism, the expression levels of nitrogen-related genes, leaf blades (6 genes) and roots (10 genes), were analyzed at the heading time using qRT-PCR. In leaf blades (Fig 4), nitrate reductase (*OsNR2*) was significantly upregulated under AN-45N treatment in both SG (1.50-fold) and ML (1.17-fold) compared to AN- 90N, while nitrite reductase (*OsNiR*) was noticeably down-regulated under 45N treatments (AS, 86%; AN, 73%) compared to 90N (100%) in ML cultivar.

**Fig 5:**
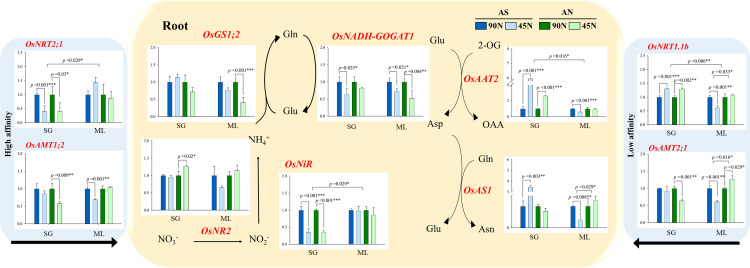
Expression of nitrogen-related genes in roots. Nitrogen form is ammonium-sulfate (AS) and ammonium-nitrate (AN), and application rate was 45 (50%) and 90 kg ha^−1^ (100%, standard fertilization rate). Mean difference between treatments was employed with Tukey’s test, if <  0.05, and T-test was used for the difference between cultivars.

Glutamine synthetase (*OsGS1;1*) and glutamine 2-oxoglutarate aminotransferase (*OsFD-GOGAT*) were similarly upregulated under AN-45N in both cultivars; SG (1.75 and 1.66 times) and ML (1.56 and 1.09 times), respectively. In roots (Fig 5), ammonium transporter (*OsAMT2;1* and *OsAMT1;2*) were significantly downregulated under SG-AN-45N (36% and 42% reduced) and ML-AS-45N (39 and 31% decreased).

The expression of nitrate transporter (*OsNRT1.1b*) was significantly upregulated in SG-AS-45N (1.31-fold) and SG-AN-45N (1.30-fold), while *OsNRT2.1* was notably decreased under these treatments (59% decreased). Other nitrogen-related genes such as *OsNR2* and *OsNiR* were also downregulated under 45N treatments, with more severely affected in SG than ML. In contrast, *OsGS1;2* and *OsNADH-GOGAT1* were remarkably downregulated at 45N treatments (18 to 59% reduced), with greater decline in ML. Interestingly, aspartate aminotransferase (*OsAAT2*) and asparagine synthetase (*OsAS1*) were highly upregulated in SG-AS-45N (5.95 and 3.43 times), while significantly downregulated in ML-AS-45N (43% and 62% reduced).

### Evaluation of nitrogen use efficiency under different N forms and rates

**Fig 6:**
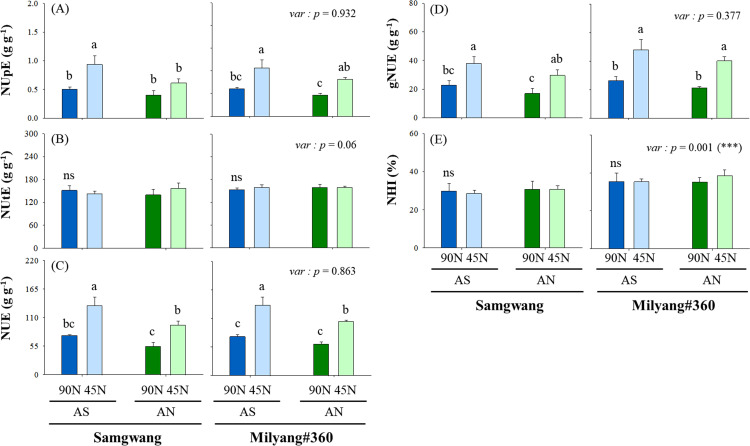
Indics of nitrogen use efficiency. Nitrogen form is ammonium-sulfate (AS) and ammonium-nitrate (AN), and application rate was 45 (50%) and 90 kg ha^−1^ (100%, standard fertilization rate). Mean difference between treatments was employed with Tukey’s test, if <  0.05, and T-test was used for the difference between cultivars.

Overall, NUE parameters (NUpE, NUE, and gNUE) did not display significant differences between two cultivars and N forms (Fig 6A–E).

However, N rate remarkably affected NUpE, NUE, and gNUE, with 45N treatments showing higher efficiency compared to 90N. Specifically, NUpE in 45N treatments was 44% (AS) and 33% (AN) higher in SG compared to 90N, and, furthermore, NUE increased by 43% (AS-45N) and 42% (AN-45N). Similar trends were also observed in ML, with AS-45N indicating a 44% increase in NUpE and 45% NUE. Grain nitrogen use efficiency (gNUE) was also significantly higher in the 45N treatments. In contrast, nitrogen utilization efficiency (NUtE) and nitrogen harvest index (NHI) were not affected by nitrogen form, rate and cultivar.

### PCA analysis

**Fig 7:**
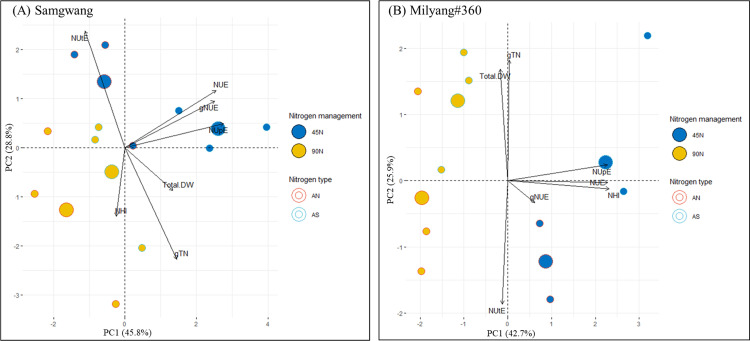
Principal component analysis (PCA) of nitrogen use efficiency traits and nitrogen fertilization.

Principal component analysis (PCA) was performed to evaluate the relationship between NUE variables and nitrogen fertilization treatments (Fig 7).

PCA revealed that the 45N and 90N treatments were distinctly separated onto a loading plot. The SG-AS-45N was closely associated with NUpE, NUE, and gNUE, suggesting a positive correlation between these variables. In ML, AS-45N was also linked to NUpE and NHI, while total dry weight and grain total nitrogen (gTN) were closely related to 90N treatment. These results indicate that nitrogen form and rate are differently responsible for biomass production and nitrogen use efficiency in both cultivars.

## Discussion

This study investigated the effects of different nitrogen (N) forms and rates on photosynthetic traits, N metabolism and nitrogen use efficiency (NUE) in two cultivars with contrasting N efficiencies; Samgwang (SG, normal N-efficient) and Milyang#360 (ML, high N-efficient). The findings provide valuable insights into optimizing nitrogen fertilization for improved rice productivity while mitigating environmental impacts.

### Rice growth, photosynthesis and soluble carbohydrates

Nitrogen fertilization is the important factor determining growth and development of crop plants, and subsequently ensuring crop productivity [30]. Based on N physiology and growth responses between rice cultivars, Samgwang (SG) and Milyang#360 (ML), were selected to evaluate CN metabolism and NUEs with different N form and rate. Overall, it was observed that ML revealed greater grain weight compared to SG despite lower tillering and shoot dry weight along with N application, and ammonium sulfate (AS) was more effective (Fig 1). Optimal N fertilization positively contributes to the enhancement of crop productivity [31,32], and difference in N forms, NO_3_^−^ and NH_4_^ + ^, was not responsible for biomass accumulation in rice [33,34]. This result aligns with previous observation [5], which suggests that AS tends to contribute to better nitrogen retention in the soil due to reduced nitrogen losses. Accordingly, our finding for greater grain weight in ML might provide a hint to improve physiological N availability under limited N fertilization. Photosynthesis is a biochemical process which is finely regulated by N source conditions [35], and showed no significant difference between cultivars affected by different N form and rate (Fig 2). This was in agreement with previous studies that N source had no noticeable effect on the photosynthesis parameters in rice [34,36,37]. In contrast, Xu et al [38] reported that ammonium or ammonium-nitrate N form contributed to greater photosynthesis compared to nitrate-only form in rice. The lack of significant differences in photosynthetic traits in ML, regardless of N form, suggests that high N-efficient cultivars may have more robust mechanisms for maintaining photosynthetic efficiency even under varying nitrogen treatments. This is consistent with previous reports that nitrogen-efficient plants often exhibit better regulation of photosynthesis and carbon-nitrogen (CN) metabolism [12,25]. N-limited conditions largely accumulate soluble sugars [11,39] and starch [40]. Soluble carbohydrates, sugar and starch, showed opposite tendency between cultivars under both N forms, especially in roots (Fig 3). The 45N-grown ML revealed significant decrease in abundance of soluble sugar and starch in roots. N deficiency greatly restricts amino acid biosynthesis as the result derived from the accumulation of cellular intermediates including soluble sugars [11,41]. Accordingly, our observation, indicating the differences in soluble sugar and starch accumulation between cultivars, suggests the distinct metabolic strategies employed by N-efficient and N-inefficient rice. ML, showing higher sugar and starch levels in leaves, is likely to be more adaptive at storing assimilated carbon, which can be remobilized during grain filling, leading to higher grain yields, and this is closely linked with the hypothesis that efficient partitioning of carbohydrates is a key determinant of NUE in high N-efficient cultivars [14].

### Nitrogen metabolism and NUEs

N metabolism is strongly affected by diverse N managements due to the differences in N absorption mechanism [42] and utilization efficiency of N fertilizers [43]. The expression analysis of nitrogen metabolism-related genes provided insights into molecular mechanisms underlying the differences in NUE between SG and ML. The upregulation of key nitrogen assimilation genes, such as nitrate reductase (*OsNR2*) and glutamine synthetase (*OsGS1;1*), in the low-N (45N) treatments suggests that reduced nitrogen input stimulates nitrogen metabolism to optimize nitrogen utilization. This is particularly evident in AN, where leaf blade in both SG and ML showed significant increases in gene expression. Thus, AS might be more effective in promoting nitrogen assimilation pathways than AN, and these findings are consistent with earlier studies that have shown the crucial role of N-assimilating enzymes in regulating nitrogen uptake and utilization efficiency [44,45]. However, it is important to note the upregulation of *OsNRT1.1b* and downregulation of *OsAMT1;1* and *OsNRT2.1*, particularly in the 45N treatments. This result proposes two possibilities that 45 kg N employed in this study didn’t cause low N condition to rice plant, while may induce low affinity nitrogen transporters to enhance N uptake efficiency. The contrasting responses of SG and ML in terms of gene expression further emphasize the genetic differences in nitrogen uptake and utilization strategies between low and high N-efficient cultivars. Additionally, the upregulation of nitrogen metabolism-related genes such as *OsNR2*, *OsGS1;1*, *OsFD-GOGAT*, *OsAS2*, in leaf blades seems to be linked with the transport of amino acids. Amino acids are major forms of remobilization from leaves to grain in rice and wheat [46,47]. Accordingly, our observation demonstrates that lower N input, not extremely N deficit, contributes N-containing metabolites such as amino acids in leaf blades.

Indeed, nitrogen rate significantly affected NUE, with lower nitrogen inputs (45N) leading to higher NUE, nitrogen uptake efficiency (NUpE), and grain nitrogen use efficiency (gNUE) in both cultivars. This is a critical finding, as it suggests that reducing N input doesn’t necessarily lead to yield loss, and, instead, can contribute to better nitrogen utilization, reducing environmental impacts such as nitrate leaching and greenhouse gas emissions [3]. The PCA analysis further supports this by showing that NUE traits were positively correlated with the 45N treatments of ammonium sulfate (AS), indicating that moderate N application can optimize NUE and improve crop sustainability.

## Conclusions

This study demonstrates the significant impact of nitrogen forms and rates on the growth, nitrogen metabolism and nitrogen use efficiency (NUE) of two contrasting rice cultivars, Samgwang (low N-efficient) and Milyang#360 (high N-efficient). Ammonium sulfate (AS) was more effective in promoting growth and NUE in both cultivars, particularly in the high N-efficient Milyang#360 (ML). AS improved photosynthetic traits, nitrogen assimilation, and carbohydrate accumulation, contributing to better overall growth and grain yield compared to ammonium nitrate (AN). High N-efficient cultivar (ML) can improve photosynthetic efficiency and regulate nitrogen metabolism more effectively under varying nitrogen conditions. This implies the importance of selecting nitrogen-efficient cultivars to optimize nitrogen use in rice production. The upregulation of nitrogen-related genes, such as nitrate reductase (*OsNR2*) and glutamine synthetase (*OsGS1;1*), was essential for enhancing NUE under low N. Low N input (45N) can enhance nitrogen use efficiency without compromising yield. Overall, these findings contribute to a deeper understanding of how nitrogen management strategies can be tailored to improve NUE in rice production. Further research should be explored under field conditions to verify CN metabolism, NUE and yield of rice with different N doses.

## Supporting information

S1 Data Raw data set.
(XLSX)
